# Supplementary material for: Inhibition of O-GlcNAc transferase activates type I interferon-dependent antitumor immunity by bridging cGAS-STING pathway
Source: eLife. 2024 Oct 4;13:RP94849. doi: 10.7554/eLife.94849 (PMC11452177; doi:10.7554/eLife.94849)
Supplement: Supplementary file 1. — (a) Primer sequences for genotype. (b) Related to Experimental Procedures. Primer sequences for RT-PCR. (c) Related to CRISPR/Cas9. Primer sequences for molecular cloning. [file elife-94849-supp1.docx]

**Supplementary file 1a. Primer sequences for genotype**

| Genes | Sequence | | Size |
| --- | --- | --- | --- |
| *Ogt^fl/fl^* | Forward CATCTCTCCAGCCCCACAAACTG | | WT 332 bp, |
|  | Reverse GACGAAGCAGGAGGGGAGAGCAC | | Mutant 487 bp |
| Villin-Cre | WT Forward TATAGGGCAGAGCTGGAGGA | | WT 182 bp, |
| (*△IEC*) | Mut Forward AGGCAAATTTTGGTGTACGG | | Mutant 150 bp |
|  | Common Rev GCCTTCTCCTCTAGGCTCGT | |  |
| *Apc^min^* | WT Forward GCCATCCCTTCACGTTAG | | WT 619 bp, |
|  | Mut Forward TTCTGAGAAAGACAGAAGTTA | | Mutant 320 bp |
|  | Common Rev TTCCACTTTGGCATAAGGC |  | |

**Supplementary file 1b. Related to Experimental Procedures. Primer sequences for RT-PCR**

| Genes | Forward | Reverse |
| --- | --- | --- |
| Mouse *Ifna4* | CCTGTGTGATGCAGGAACC | TCACCTCCCAGGCACAGA |
| Mouse *Ifnb1* | ATGAGTGGTGGTTGCAGGC | TGACCTTTCAAATGCAGTAGAGTCA |
| Mouse *Ifng* | TCAAGTGGCATAGATGTGGAAGAA | TGGCTCTGCAGGATTTTCATG |
| Mouse *Il1a* | GCACCTTACACCTACCAGAGT | AAACTTCTGCCTGACGAGCTT |
| Mouse *Il1b* | CTCATTGTGGCTGTGGAGAAG | ACCAGCAGGTTATCATCATCAT |
| Mouse *Il6* | AGCTGGAGTCACAGAAGGAG | AGGCATAACGCACTAGGTTT |
| Mouse *Il10* | CCCTTTGCTATGGTGTCCTT | TGGTTTCTCTTCCCAAGACC |
| Mouse *Il12a* | GAGGACTTGAAGATGTACCAG | TCCTATCTGTGTGAGGAGGGC |
| Mouse *Tnfa* | GTCAGGTTGCCTCTGTCTCA | TCAGGGAAGAGTCTGGAAAG |
| Mouse *Cxcl10* | CCTGCCCACGTGTTGAGAT | TGATGGTCTTAGATTCCGGATTC |
| Mouse *Isg15* | TGGAAAGGGTAAGACCGTCCT | GGTGTCCGTGACTAACTCCAT |
| Mouse *Mx1* | GGGGAGGAAATAGAGAAAATGAT | GTTTACAAAGGGCTTGCTTGCT |
| Mouse *Actb* | AGGGCTATGCTCTCCCTCAC | CTCTCAGCTGTGGTGGTGAA |
| Human *IFNB1* | CATTACCTGAAGGCCAAGGA | CAATTGTCCAGTCCCAGAGG |
| Human *ISG15* | CTGAGAGGCAGCGAACTCAT | AGCATCTTCACCGTCAGGTC |
| Human *MX1* | AGAGAAGGTGAGAAGCTGATCC | TTCTTCCAGCTCCTTCTCCTG |
| Human *CXCL10* | CTCCAGTCTCAGCACCATGA | GCTCCCCTCTGGTTTTAAGG |
| Human *GADPH* | ATGACATCAAGAAGGTGGTG | CATACCAGGAAATGAGCTTG |

**Supplementary file 1c. Related to CRISPR/Cas9. Primer sequences for molecular cloning**

| Genes | Forward | Reverse | |  |
| --- | --- | --- | --- | --- |
| *Ogt* gRNA#1 | CACCGTGCCCACGGAAGACGCCATC | | AAACGATGGCGTCTTCCGTGGGCAC | |
| *Ogt* gRNA#2 | CACCGGCTCCAGATGGCGTCTTCCG | | AAACCGGAAGACGCCATCTGGAGCC | |
| *mMavs* gRNA#1 | ACCGGCCGTCGCGAGGATGTCTGG | | AACCCAGACATCCTCGCGACGGCC | |
| *mMavs* gRNA#2 | CACCGGATACCCTCTCCTAACCAGC | | AACGCTGGTTAGGAGAGGGTATCC | |
| *mCgas* gRNA | CACCGATATGGAAGATCCGCGTAGA | | AAACTCTACGCGGATCTTCCATATC | |
| *mSting* gRNA | CACCGGCTGGATGCAGGTTGGAGTA | | AAACTACTCCAACCTGCATCCAGCC | |
| *hcGAS* gRNA | CACCGAAGTGCGACTCCGCGTTCAG | | AAACCTGAACGCGGAGTCGCACTT | |
| *hSTING* gRNA | CACCGGGATGTTCAGTGCCTGCGAG | | AAACCTCGCAGGCACTGAACATCC | |
